# Selfish Play Increases during High-Stakes NBA Games and Is Rewarded with More Lucrative Contracts

**DOI:** 10.1371/journal.pone.0095745

**Published:** 2014-04-24

**Authors:** Eric Luis Uhlmann, Christopher M. Barnes

**Affiliations:** 1 Management and Human Resources Department, HEC Paris, Jouy-en-Josas, France; 2 Department of Management and Organization, Foster School of Business, University of Washington, Seattle, Washington, United States of America; Universidad Carlos III de Madrid, Spain

## Abstract

High-stakes team competitions can present a social dilemma in which participants must choose between concentrating on their personal performance and assisting teammates as a means of achieving group objectives. We find that despite the seemingly strong group incentive to win the NBA title, cooperative play actually *diminishes* during playoff games, negatively affecting team performance. Thus team cooperation decreases in the very high stakes contexts in which it is most important to perform well together. Highlighting the mixed incentives that underlie selfish play, personal scoring is rewarded with more lucrative future contracts, whereas assisting teammates to score is associated with reduced pay due to lost opportunities for personal scoring. A combination of misaligned incentives and psychological biases in performance evaluation bring out the “I” in “team” when cooperation is most critical.

## Introduction

“When you score a goal, or hit a three, or get a touchdown, you don't do it for yourself, you do it for the team ’cause the name on the front of the shirt is more important than the one on the back.”

– Herb Brooks, coach of the 1980 U.S. Olympic hockey team

This success of any team depends on the commitment of its members to not only excel personally, but also aid one another. However, a trade-off inevitably exists between concentrating on one's personal performance and assisting teammates as a means of achieving group objectives (i.e., engaging in *backing up behavior*; [1]–[3]). Organizations can structure incentives to reward either team members' individual achievements (*individual incentives*), collective achievements (*group incentives*) or both (*mixed incentives*) [4]–[8]. Although the use of mixed incentives is intuitively appealing, they can create a social dilemma [9]–[12] which agents tend to resolve by maximizing their own performance and individual rewards at the expense of cooperative behavior [13]–[14].

Professional sports teams represent a fascinating case of mixed incentives because teams complete fiercely for prestigious group honors (e.g., the National Basketball Association title, Super Bowl Championship), yet reward players financially based on evaluations of their individual performance. Formal incentives that reward group performance vary between games, and in many sports are especially strong during playoff games, which represent an opportunity to win the coveted league title. This should by design lead to greater teamwork (i.e., increased backing-up behavior) as players seek to enhance their collective chances of winning a prize that for many represents a lifelong dream.

But at the same time, prior research and theory provide reasons to predict that high-stakes games are actually characterized by *less* cooperative play than comparatively less important games. Despite all the rhetoric exhorting athletes to play for the team rather than themselves, psychological biases may lead salient indices of individual performance (such as points scored) to be overweighed in evaluations of player quality and economic value relative to backing-up behavior (such as assisting others to score). Research on correspondent inferences demonstrates that social perceivers automatically attribute behavior to the agent's underlying traits and fail to consider the role of the situation and surrounding context [15]–[16]. One such often ignored contextual factor may be the team passing that set up a player to score. At the same time, inferences about underlying characteristics are most readily elicited by information and events that are highly salient [17]–[18], and points scored are clearly more attention-grabbing than passes and assists. Thus, players' personal prestige and financial compensation may be more closely linked to their individual scoring tally than to their contributions to points scored by teammates, a perverse incentive that could be strengthened by the increased public attention attracted by high-stakes games.

Indeed, important games shine the spotlight of international attention not only on the team as a whole but also on each individual player, providing increased opportunities to have one's talents recognized by fans, sponsors, and employers. Players may respond by adopting a noncooperative strategy aimed at increasing their personal prestige and economic value by maximizing salient indices of their individual performance [19]. Absent any guarantee teammates will adopt a cooperative strategy or reciprocate backing-up behavior, the most individually rational response to the mixed-incentives social dilemma posed by a high-stakes game may be to defect [20]–[21] and take advantage of any cooperative play by others to increase one's own individual scoring tally. Thus, psychological biases in evaluations of performance and the unintended consequences of group rewards may conspire to reduce team cooperation and success, ironically under those very conditions in which working together is most crucial.

## Methods

Our data were drawn from statistics made public by the National Basketball Association (NBA). We examined player and team behavior and performance across all 30 teams from the 2004–2005 through the 2012–2013 seasons. These data included our measure of cooperative team play, the ratio of field goals (a basket scored on anything other than a free throw) to cases in which players assisted a teammate to score (assists made). A low number of assists per field goal indicates a lack of cooperation among team members, whereas a high number of assists per field goal indicates a high level of team play. Notably, operationalizing cooperative play as assists *relative* to field goals helps control for the pace of the game as well as the intensity of the defense. Different offensive pace and defensive intensity may lead to fewer field goals scored in the playoffs than in the regular season. By accounting for the number of field goals scored, we account for these differences. To further account for defensive intensity, we included turnovers as a control variable; if aggressive defenders manage to steal possession of the ball from their opponents, such steals are reflected in turnover statistics. We included data from both the regular season as well as the playoffs when teams are competing for the championship (i.e., our high stakes context). One team did not make the playoffs in the duration of the sample. Of the remaining 29 teams, only 16 were included in the playoffs in any given year in the 9 year sample. This provides a final sample of 144 team-years for comparing team cooperation in the regular season vs. in the playoffs.

In order to test the effects of cooperative play on team performance, we further captured the number of wins both in the playoffs and the regular season, as officially recorded by the NBA. For the regular season, this included all 30 teams for all 9 seasons, producing a sample of 270 team-years. For the playoffs, this included only the 16 teams per year that appeared in the playoffs, producing a sample of 144 team-years. We expected that cooperative play would positively predict team wins in both playoff games and during the regular season, and that there would be no differences in the effects of cooperative play between playoff and regular season games (since cooperation should contribute to team success regardless of whether it is a high-stakes or low-stakes situation). We introduce a new measure of team cooperation to the empirical literature, operationalizing the tendency to play as a team as the ratio of field goals made (a basket scored on anything other than a free throw) to cases in which players assisted a teammate to score (assists). As we lacked data on assists attempted, for equivalence we did not consider field goals attempted in our analyses. To account for non-independence of observations due to the fact that some teams have more success over time (i.e., team performance is nested within teams), we conducted a multilevel analysis using hierarchical linear modeling [22]. This entailed nesting team-years (Level 1) within teams (Level 2). There were no substantive variables at Level 2; Level 2 was only included to account for the non-independence of team-years. This approach was supported by an ICC(1) analysis, which indicated that 25% (*p*<.01) of the variance in regular season team-year performance and 14% (*p*<.05) of the variance in playoff team-year performance was accounted for by the team level of analysis.

To test the effect of solo scoring and assists to team members on individual compensation, we examined labor contracts signed by individual NBA players following the 2003–2004 and 2004–2005 seasons. As collecting individual salary data for every NBA player was labor intensive we stopped after obtaining a sufficient sample (*N* = 131 players across two full years) to test our theoretical hypotheses regarding changes in compensation across time. In order to detect a moderate-to-small effect of .30 with a *p* value of .05, we needed a sample of at least 80 players. One year of players with available salary data was less than 80 (57 players for the 2003–2004 season), so we gathered data for a second year (74 players for the 2004–2005 season). The sum of these two years was 131, which was greater than the 80 needed to detect a moderate-to-small effect. We excluded players on rookie contracts, which are not determined by previous NBA play. This resulted in a final sample of 131 NBA players along with their salary from their previous contract and their officially tracked variables of number of field goals scored, assists to team members (passes to other players which result in a field goal scored by that player), and the control variables of minutes played, turnovers, and whether or not the player played the “center” position (which commands a salary premium). This analytic approach allowed us to examine the extent to which each player's behavior over the course of the season contributed to changes in his financial compensation, controlling for past salary and potential third variables. The average age of the players in our sample was 29.00 years (*SD* = 4.15 years), and they averaged 6.85 years in the league (*SD* = 3.42 years) and received an average annual compensation of $5,228,701.87 (*SD* = $5,178,327.55).

## Results

Our first analysis was the effect of high stakes contexts on the tendency to play as a team. For this analysis we conducted a paired sample t-test comparing assists per field goal in the regular season versus the playoffs for each given team year. This test confirmed that the ratio of assists per field goal made in the regular season (*M* = .59) was higher than assists per field goal made in the playoffs (*M* = .54), with the difference being highly significant, *t*(143) = 10.39, *p*<.001. This indicates that, as hypothesized, cooperative team play declined significantly in the high-stakes context of the playoffs in comparison to the regular season. Although the difference between .59 and .54 in assists per field goal between the regular season and playoffs may seem small at first glance, the standard deviation of this statistic was only .038, such that our effect was in fact over one standard deviation. Further, at the highest levels of competition all differences matter, and (as reported below), the ratio between assists and field goals significantly predicts important team wins.

Our second analysis was the effect of team cooperation on performance. As shown in Table 1, the hierarchical linear modeling analyses revealed that in the playoffs, cooperation had a significant positive relationship with team performance, *B* = 15.31, *p*<.05. In the regular season a similar pattern of results emerged but failed to reach statistical significance, *B* = 36.99, *p*>.10. Thus, our hypothesis that team cooperation would be positively linked to team wins received strong empirical support in the context of the playoffs, but (unexpectedly) not for regular season games. There was however no significant interaction between the playoffs and regular season in terms of the relationship between cooperation and wins (and as noted earlier no such interaction was expected).

**Table 1:** Effects of Team Cooperation on Team Performance.

	Regular Season			Playoffs		
	Coefficient	Standard Error	*t* ratio	Coefficient	Standard Error	*t* ratio
Intercept	40.56	1.28	31.86**	4.93	0.50	9.90**
Cooperation	36.98	28.75	1.29	15.31	7.42	2.06*

Regular season: Level 2 N = 30 teams, Level 1 N = 270 team-years.

Playoffs: Level 2 N = 29 teams, Level 1 N = 144 team-years.

*p<.05.

**p<.01.

Our final analysis was the effects of field goals and assists to team members on individual compensation. As seen in Table 2, field goals had a significant positive effect on subsequent salary, *B* = 22044.55, *p*<.001. In contrast, assists to team members had a marginally significant negative effect on subsequent salary, *B* = −6116.69, *p* = .08. However, a bias-corrected bootstrap 95% confidence interval analysis indicated that assists to team members had a significant negative indirect effect on subsequent salary through the mediator of field goals scored (95% confidence interval: lower bound of −11081.03, upper bound of −1701.41). Thus, assists to team members predicted a lower future salary due to the fact that this cost the player opportunities to personally score. In financial terms, every field goal personally scored by a player increases his salary by $22,044.55, and every assist he provides to another player decreases his salary by $6,116.69.

**Table 2:** Individual Behavior and Compensation.

	Total Effect			
Predictor	Outcome	Coefficient	Standard Error	T value
Assisting Team Members	Salary	−6116.69	3504.52	−1.75†

	Direct Effects			
Predictor	Outcome	Coefficient	Standard Error	T value
Assisting Team Members	Solo Scoring	−0.24	0.06	−3.93**
Solo Scoring	Salary	2204.55	4875.43	4.52**
Assisting Team Members	Salary	−.931.24	3455.95	−0.27

Covariates	Outcome	Coefficient	Standard Error	T value
Previous Salary	Salary	0.18	0.07	2.61*
Minutes Played	Salary	−1363.32	853.49	−1.6
Center Position	Salary	2043604.1	764599.61	2.67**
Turnovers	Salary	13324.79	13468.22	0.99

	Indirect Effect			
			95% Confidence Interval
Predictor	Mediator	Outcome	Lower Bound	Upper Bound
Assisting Team Members	Solo Scoring	Salary	−11081.03	−1701.41

N = 131.

† *p*<.10.

* *p*<.05.

** *p*<.01.

## Discussion

Despite the presence of the extraordinary opportunity to win the national title, we find that cooperative team play actually *diminishes* during NBA playoff games. This demonstrates for the first time that team cooperation can decrease in the very high stakes contexts in which it is most important to perform well together, and even under conditions designed to reward group performance. Shedding light on the motives that underlie selfish play, personal scoring in the NBA is rewarded with more lucrative future contracts, whereas assisting teammates to score is actually associated with *reduced* future pay due to lost opportunities for personal scoring. This bias in performance evaluation creates a mixed-incentives social dilemma [23], in which players must choose between maximizing their personal scoring tally and market value vs. assisting teammates as a means of achieving the collective goal to win games. Our results indicate that in high-stakes team competitions such as the NBA playoffs, which attract increased attention from fans, sponsors, and employers, this dilemma is especially likely to be resolved with defection and noncooperative play.

An important question for future research is whether players are motivated by a desire to receive a more lucrative contract or whether monetary pay is better considered a proxy for more intangible rewards, such as public acclaim and prestige. Regardless of whether the rewards of noncooperative play are primarily material or psychological, our analyses make it clear such behavior is especially likely in high-stakes games and is rewarded by increases in individual level financial compensation.

Future research should also examine whether overweighing points scored relative to assists in players' financial compensation reflects biases on the part of the team's management or the team's fans. The negative effect of assists on future pay is truly remarkable given widespread rhetoric from coaches and owners regarding the importance of team play, and that assisting others to score is just as quantifiable and routinely measured as points directly scored. Notably, however, professional athletic teams exist not only to win games and titles, but also to attract viewership, fill stadiums, and sell merchandise. Owners and coaches may very well understand the importance of backing-up behavior to team success, but value players who act to maximize their individual scoring tally precisely because they know fans will do so.

The present study compared NBA basketball games that varied in their objective professional stakes, specifically playoff games featuring an opportunity to win the title vs. regular season games. However, even within the regular season NBA basketball games are likely to vary in subjective importance for both fans and players. For instance, derby matches between bitter rivals are characterized by more intense interest from fans and more aggressive tactics by players, suggesting a high level of psychological importance is placed on the outcome [24]. If so, then derby matches may likewise be characterized by less cooperative play, a possibility worth examining in future research.

Of further interest is the extent to which cooperative and noncooperative behaviors are contagious within the social network of a team [25]–[27]. We hypothesize that noncooperative or “selfish” play is more likely to spread from player to player than cooperative play. An environment in which teammates fail to reciprocate backing-up behavior should lead players to adopt a noncooperative strategy themselves to avoid receiving the negative “sucker's payoff” [28]. In contrast, observing ones teammates engage in frequent backing up behavior may only increase the temptation to defect [29]–[30] and maximize one's own scoring opportunities.

Although our analyses reveal clear benefits of noncooperative play at an individual level, there are almost certainly limits to the effectiveness of such a strategy. Some team members may censure noncooperative play by choosing not to back up selfish players by creating scoring opportunities for them [31]. In extreme cases, a reputation as someone who plays for himself rather than the team may damage a player's value in the eyes of peers, fans, and coaches. Thus, reputational concerns and costly punishment by teammates likely circumscribe individuals' willingness to adopt noncooperative strategies.

Finally, there are reasons to anticipate both between-culture and within-culture differences in responses to the dilemma created by multiple incentive reward structures. In contrast to members of individualistic cultures like the United States, people from collectivistic cultures are less likely to discount contextual influences on performance [32]–[33], tend not to socially loaf during group endeavors [34], and view helping coworkers as an opportunity rather than a burden [35]. This raises the possibility that players in nations such as China, Japan, and India may not reduce their backing-up behavior during important games. Also, that women are more relationally oriented than men [36] suggests that even within the United States, high-stakes games in women's sports leagues may not be associated with diminished levels of team cooperation.

In spite of numerous inspiring quotes, speeches, and anecdotes lauding the virtues of team play, the temptation often exists to refrain from supporting one's teammates and pursue personal achievement instead. The present research indicates that such noncooperative strategies are encouraged by biases in performance evaluation that reward salient individual achievements over cooperative contributions, and can ironically be exacerbated by team rewards. We look forward to future research on the psychological motivations and situational incentives that bring out the “I” in “team.”
